# Metabolic Syndrome and Parkinson’s Disease: Two Villains Join Forces

**DOI:** 10.3390/brainsci15070706

**Published:** 2025-06-30

**Authors:** Lucas Udovin, Sofía Bordet, Hanny Barbar, Matilde Otero-Losada, Santiago Pérez-Lloret, Francisco Capani

**Affiliations:** 1 Centro de Altos Estudios en Ciencias Humanas y de la Salud (CAECIHS), Universidad Abierta Interamericana—Consejo Nacional de Investigaciones Científicas y Técnicas (UAI-CONICET), Buenos Aires C1270AAH, Argentina; lucas2304@hotmail.com (L.U.); sofia.bordet@gmail.com (S.B.); hannyjrb@gmail.com (H.B.); molly1063@gmail.com (M.O.-L.); 2 Centro de Investigaciones en Psicología y Psicopedagogía (CIPP), Facultad de Psicología y Psicopedagogía, Pontificia Universidad Católica Argentina (UCA), Buenos Aires C1107AFB, Argentina; 3 Instituto Universitario de Ciencias de la Salud, Fundación H.A. Barceló, Buenos Aires 1127, Argentina; splloret@gmail.com; 4 Departamento de Fisiología, Facultad de Medicina, Universidad de Buenos Aires, Buenos Aires C1121ABG, Argentina; 5 Facultad de Medicina, Universidad Autónoma de Chile, Santiago 8900000, Chile

**Keywords:** Parkinson’s disease, metabolic syndrome, neurodegeneration, genetic traits

## Abstract

**Background:** Metabolic syndrome and Parkinson’s disease have common pathophysiological denominators. This study aimed to investigate how metabolic syndrome contributes to Parkinson’s disease progression, as well as the genetic traits shared by PD and MetS. **Methods:** Four hundred and twenty-three newly diagnosed drug-naïve PD patients were analyzed from the Parkinson’s Progression Markers Initiative (PPMI) database. We compared longitudinal changes in the total and subscale scores of the Movement Disorder Society-Unified Parkinson’s Disease Rating Scale (MDS-UPDRS) between PD patients with and without metabolic syndrome over a five-year follow-up. We assessed the frequency of PD-associated genetic variants in both groups. **Results:** At baseline, Parkinson’s patients with MetS were typically men (*p* < 0.01) and older (*p* = 0.04), with a higher Hoehn and Yahr score (*p* = 0.01) compared with their counterparts without MetS. They showed higher Movement Disorder Society-Unified Parkinson’s Disease Rating Scale (MDS-UPDRS) total scores at baseline and in follow-up years 2, 3, 4, and 5 (all *p*-values < 0.05) as analyzed by the Generalized Estimating Equation model. These differences were primarily driven by elevated motor scores (MDS-UPDRS Part III) (*p* < 0.01). MetS was associated with a higher frequency of the ZNF646.KAT8.BCKDK_rs14235 variant and a lower frequency of the NUCKS1_rs823118 and CTSB_rs1293298 variants. **Conclusions:** PD patients with MetS had worse motor symptomatology. Both conditions appear to share genetic susceptibility, involving genes related to lipid metabolism (BCKDK), autophagy and inflammation (CTSB), and chromatin regulation (NUCKS1).

## 1. Introduction

Parkinson’s disease (PD) is the second most common neurodegenerative disorder globally, after Alzheimer’s disease [1]. Cardinal traits of PD are progressive motor symptoms like resting tremor, muscle rigidity, bradykinesia, postural instability, and gait freezing [1], and non-motor symptoms like autonomic, cognitive, and mood disturbances [2]. PD is defined by the accumulation of misfolded α-synuclein protein—Lewy bodies—in neurons, which leads to the degeneration of dopaminergic neurons in the substantia nigra [1,3]. Both genetic predisposition and environmental factors contribute to pathogenic processes, including mitochondrial dysfunction, oxidative stress, protein aggregation, and chronic neuroinflammation [3]. The resultant neurodegenerative cascade is multifactorial: mitochondrial dysfunction, disrupted calcium homeostasis, synaptic alterations, and chronic neuroinflammation contribute to PD progression [3]. PD is a complex disorder with both central and peripheral manifestations, potentially influenced by systemic metabolic factors.

Metabolic syndrome (MetS) is the cluster of interrelated cardiometabolic risk factors. Abdominal obesity, insulin resistance, hypertension, hyperglycemia, and dyslipidemia concur to increase cardiovascular and renal disease, stroke, and type 2 diabetes [4,5]. MetS affects roughly one-quarter of the adult population and is associated with increased all-cause mortality [4,5]. On a molecular level, MetS is characterized by chronic metabolic disturbances with systemic oxidative stress and mild-to-severe inflammation [6]. Similar oxidative and inflammatory processes are implicated in PD pathogenesis [4,7,8], suggesting a potential pathophysiological bridge between these two conditions. Indeed, mounting evidence indicates that PD patients who also meet MetS criteria tend to go through a more severe disease than those without MetS. Clinical studies have reported that PD patients with MetS exhibit higher motor impairment and non-motor symptom burden, including worsened cognitive function, compared with their metabolically healthier counterparts [9,10,11]. One cross-sectional study found higher total scores on the Non-Motor Symptoms Scale in PD patients with MetS versus those without MetS [9]. These observations emphasize that metabolic comorbidities can modulate the clinical expression of PD.

The link between MetS and PD is biologically supported, as both conditions share key pathophysiological mechanisms. Both MetS and PD show insulin resistance, chronic inflammation, and oxidative stress, which can synergistically promote neuronal dysfunction and death [7,8]. Obesity-driven peripheral inflammation in MetS may trigger neuroinflammatory changes in the brain, exacerbating dopaminergic neuron loss in PD [8]. Likewise, sustained insulin resistance—a hallmark of MetS—has been observed in the brains of PD patients, where it may impair insulin signaling needed for neuronal survival and amplify α-synuclein toxicity [7]. Mitochondrial dysfunction is another common denominator: metabolic syndrome can induce mitochondrial abnormalities in peripheral tissues, and PD neurons are particularly vulnerable to deficits in mitochondrial energy production, linking metabolic stress and neurodegenerative processes [3,12]. These convergent mechanisms provide a compelling scientific rationale for the epidemiological and clinical association between MetS and PD. However, despite the growing recognition of this interplay, the precise molecular and genetic connections between MetS and PD remain largely unexplored. To date, most research has focused on epidemiological associations, leaving a gap in understanding whether shared genetic variants contribute to both metabolic and neurodegenerative pathways [13]. Few studies have systematically evaluated whether specific PD-associated single nucleotide polymorphisms (SNPs) differ in frequency among patients with or without MetS, or whether these variants modify disease severity.

Clinical and genetic evidence supports further investigation into the MetS–PD bond. The importance of the early identification and management of metabolic dysfunction in PD patients seems supported by the MetS acceleration of PD progression or exacerbation of PD symptoms. From a genetic standpoint, any shared genetic susceptibilities or divergent variant profiles could reveal novel biological pathways linking metabolic regulation and neurodegeneration. This study combines a longitudinal clinical analysis with a genetic investigation to clarify how MetS and PD “join forces” to address these gaps. We analyzed a large cohort of newly diagnosed drug-naïve PD patients to evaluate the impact of coexistent MetS on the severity and progression of motor and non-motor symptoms. In parallel, we compared the frequencies of PD-related gene variants in patients with and without MetS to uncover potential common genetic underpinnings of the two conditions. This dual approach, integrating clinical outcomes with genetic data, provides a novel and comprehensive perspective on the interaction between MetS and PD. Our study aims to advance the understanding of why and how (genetics and clinical consequences) these two medical villains join forces, warranting the consideration of metabolic status in PD research and therapy.

## 2. Materials and Methods

### 2.1. Study Participants

This study analyzed data from 423 newly diagnosed drug-naïve Parkinson’s disease (PD) patients included in the Parkinson’s Progression Markers Initiative (PPMI) cohort [1]. Eligible participants met the following inclusion criteria: (1) PD diagnosis within the past two years, (2) Hoehn and Yahr stage I or II at enrollment, (3) dopaminergic deficit confirmed by dopamine transporter (DAT) single-photon emission computed tomography (SPECT), and (4) no need for antiparkinsonian medication within six months of baseline evaluation. The PPMI study was approved by the institutional review boards of all participating centers, and written informed consent was obtained from all participants for data sharing and research use [1].

### 2.2. Study Assessments

This was a longitudinal cohort study that retrieved and analyzed clinical and genomic data from the PPMI database. Participants were evaluated at baseline and annually for five years. PD symptom progression was assessed using the Movement Disorder Society-Unified Parkinson’s Disease Rating Scale (MDS-UPDRS) [14]. This validated instrument comprises four parts: Part I assesses non-motor experiences of daily living (e.g., cognition and mood); Part II evaluates motor experiences of daily living; Part III is a motor examination performed by clinicians; Part IV assesses motor complications related to antiparkinsonian treatment. Each item is scored from 0 (normal) to 4 (severe), with higher total scores indicating larger impairment. We examined the total MDS-UPDRS score and the subscores from Parts I–IV at each annual visit. Part III motor evaluations were conducted in the practically defined “off-medication” state, as patients were untreated at study entry.

Baseline metabolic syndrome (MetS) status was defined according to the Adult Treatment Panel III (ATP III) criteria [5], requiring at least three of the following five components:

- Obesity: body mass index (BMI) > 30 kg/m^2^.
- Hyperglycemia: fasting glucose ≥ 100 mg/dL, previously diagnosed diabetes mellitus, or current use of antidiabetic medication.
- Hypertriglyceridemia: triglycerides ≥ 150 mg/dL, or prior diagnosis/treatment for elevated triglycerides.
- Low HDL cholesterol: HDL < 40 mg/dL in men or <50 mg/dL in women, or current treatment for low HDL levels.
- Hypertension: blood pressure ≥ 130/85 mmHg, previously diagnosed hypertension, or use of antihypertensive therapy.

### 2.3. Genetic Assessment in PD Patients with or Without MetS

Baseline blood samples were collected for whole-genome sequencing (WGS), performed by Macrogen Inc. (Seoul, Republic of Korea). A Covaris system was used for genomic DNA fragmentation. The outcome was processed using the Illumina TruSeq library preparation protocol, yielding paired-end libraries with an average of 300 to 400 base pairs (bp) insert size. An Illumina HiSeq X platform allowed sequencing. Raw reads were arrayed to the GRCh37 human reference genome (BWA-MEM, v0.7.13), and BAM files were sorted and deduplicated (Bamsormadup2, v2.0.87).

Base quality score recalibration and local realignment were performed with the Genome Analysis Toolkit (GATK v3.5). Variant calling was conducted using GATK HaplotypeCaller, generating variant call format (VCF) files.

Genotype data were harmonized to the GRCh38 build using BCFtools and PLINK for analysis. We focused on 72 single nucleotide polymorphisms (SNPs) previously associated with increased PD risk in a large genome-wide association study [13]. SNPs were included if they met standard quality control criteria: call rate ≥ 95%, minor allele frequency (MAF) > 1%, and Hardy–Weinberg equilibrium *p* > 0.05.

### 2.4. Statistical Analysis

Descriptive comparisons between patients with and without MetS were performed using Student’s *t*-tests (for continuous variables) and chi-square tests (for categorical variables). Longitudinal changes in clinical scores were modeled using generalized estimating equations (GEE) [15], which are appropriate for repeated-measures data. In the GEE model, the MDS-UPDRS total score was used as the dependent variable, with MetS status (yes/no) and time (in years) as primary factors. Covariates included age, sex, and the use of antiparkinsonian medication. An interaction term (MetS × Time) was included to evaluate whether symptom progression over time differed by MetS status. When a significant interaction was detected, post hoc comparisons of the model-estimated marginal means were conducted at each time point to identify significant between-group differences.

To explore genetic associations with MetS, we first tested each of the 72 candidate SNPs for association with baseline MetS status using logistic regression models adjusted for age and sex. For each SNP, three genetic models (dominant, recessive, additive) were evaluated, and the model with the lowest Akaike Information Criterion (AIC) was selected. SNPs showing a nominal association (*p* < 0.15) were included in a multivariate logistic regression to identify independent predictors of MetS. The final model was adjusted for age and sex, and statistical significance was set at *p* < 0.05. All analyses were performed using SPSS v24 (IBM Corp., Armonk, NY, USA) and R v4.2.2 (R Foundation for Statistical Computing, Vienna, Austria).

## 3. Results

### 3.1. Baseline Demographic and Clinical Characteristics

A total of 423 patients with Parkinson’s disease were analyzed, of whom 34 (8.0%) met criteria for metabolic syndrome (MetS) at baseline. Patients with MetS (PD + MetS) differed significantly from those without (PD–MetS) in several baseline characteristics (Table 1). Specifically, the MetS group was predominantly male (94% vs. 63% in PD–MetS; *p* < 0.01) and slightly older (mean age 64.9 ± 9.4 vs. 61.4 ± 9.7 years; *p* = 0.04). They also had more advanced disease: a higher proportion were Hoehn and Yahr stage II at baseline (76% vs. 54%; *p* = 0.01), whereas stage I was correspondingly less common (Table 1). Baseline MDS-UPDRS total scores were higher in PD + MetS (30.7 ± 9.6) than in PD–MetS (26.5 ± 11.2; *p* = 0.03), indicating greater overall symptom burden. In contrast, the prevalence of a family history of Parkinson’s disease did not differ between groups (24% vs. 32%, *p* = 0.38).

### 3.2. Longitudinal MDS-UPDRS Scores and Subscales

**Figure 1:**
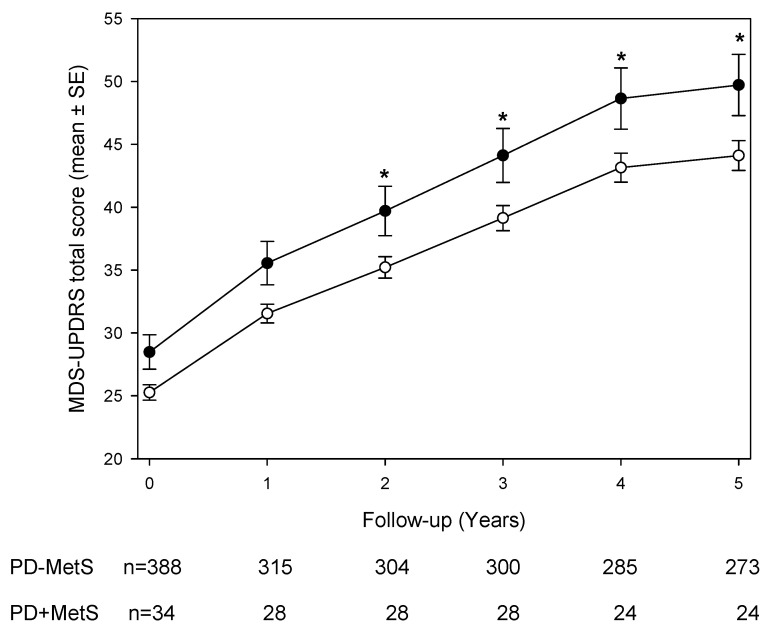
MDS-UPDRS total scores in PD patients with (-●-) or without (-○-) metabolic syndrome over the 5-year follow-up period. GEE found an overall significant between-patient group difference (*p* < 0.01). Least-squares means derived from the GEE model after including the MetS-Time interaction, adjusted by sex, age, and antiparkinsonian treatment, are shown. * *p* < 0.05 (post hoc comparisons).

Figure 1 illustrates the longitudinal course of MDS-UPDRS total scores over five years. Both groups showed progressive increases in motor scores, but PD + MetS patients consistently exhibited higher scores at each annual visit. The overall group difference was highly significant (*p* < 0.01 by generalized estimating equation [GEE] analysis). After adjusting for age, sex, and antiparkinsonian treatment, a GEE model with a MetS-by-time interaction revealed that the PD + MetS group had significantly higher MDS-UPDRS total scores than the PD–MetS group during years 2 through 5 (post hoc *p* < 0.05 for each of these time points). In contrast, at baseline and year 1, the between-group difference did not reach statistical significance (Figure 1). Subscale analyses of the MDS-UPDRS (Table 2) showed that differences were confined to specific domains. Non-motor aspects (Part I) and motor complications (Part IV) scores did not differ significantly between PD + MetS and PD–MetS at any time point (all *p* > 0.05). Motor aspects of daily living (Part II) were significantly worse in the MetS group at early visits: mean Part II scores were higher in PD + MetS at baseline (7.1 ± 4.7 vs. 5.8 ± 4.1; *p* < 0.01) and year 1 (9.4 ± 4.5 vs. 7.2 ± 5.0; *p* < 0.05), but this gap narrowed in subsequent years. The most consistent differences were observed in the motor examination subscore (Part III). PD + MetS patients had higher Part III scores at every visit. For example, at baseline the mean Part III score was 23.6 ± 7.2 in the MetS group versus 20.7 ± 9.0 in the non-MetS group (*p* < 0.01), and elevated scores persisted through year 5 (mean 35.7 ± 9.5 vs. 29.9 ± 13.6; *p* < 0.05) (Table 2). These findings indicate that patients with MetS experienced consistently worse motor function over time.

### 3.3. Genetic Association Analysis

Genetic analyses were performed in the 409 patients with complete genotype data (376 PD–MetS, 33 PD + MetS; Table 3). Three Parkinson’s-associated SNPs showed significant frequency differences by MetS status. The ZNF646.KAT8.BCKDK_rs14235 variant was significantly overrepresented in PD + MetS: 30.3% of MetS patients were homozygous for the risk allele compared to 15.6% of non-MetS patients (recessive model OR 3.06, 95% CI 1.24–7.29; *p* = 0.012). In contrast, risk alleles of NUCKS1_rs823118 and CTSB_rs1293298 were underrepresented in the MetS group. For NUCKS1_rs823118, only 6.1% of MetS patients carried two risk alleles versus 18.8% of non-MetS patients (OR 0.21, 95% CI 0.03–0.77; *p* = 0.043). Similarly, carriage of at least one CTSB_rs1293298 risk allele was less common in PD + MetS (21.2% vs. 43.8%; OR 0.35, 95% CI 0.13–0.84; *p* = 0.025). No significant differences were observed for other tested variants (e.g., GBA_N370S, COMT_rs4633). These genotype–phenotype associations (Table 3) suggest that specific genetic factors may influence the likelihood of developing metabolic syndrome in PD.

## 4. Discussion

The PPMI is a cohort study involving patients from around the world. Patients are included immediately after being diagnosed with PD and are followed for at least 5 years. Patients are assessed by certified instruments in a standardized manner. Therefore, our findings of more serious motor symptoms in PD patients with MetS are robust and might be generalizable to patients outside the study.

Recent studies suggest that MetS increases the risk of PD. Indeed, in a Korean study involving more than 17 million people, MetS significantly increased the lifetime risk of suffering from PD by 24% [16]. In the same database, Roh et al. showed that PD risk increased with the number of MetS components [17]. Waist circumference was not related to PD incidence, while high fasting glycemia, low high-density lipoprotein cholesterolemia, and hypertension increased it by 20–34%. Hypertriglyceridemia appeared to protect against PD in men. Their results have been confirmed by other studies, including a recent meta-analysis involving over 23 million participants’ data across eleven articles [18].

The evidence regarding MetS’s impact on PD severity is less abundant. Ninety-nine PD patients from Mexico—8% having MetS—showed differences in the perceptual, mood/apathy, gastrointestinal, sexual function, and miscellaneous traits of the Non-Motor Symptoms Scale [9]. No differences were found in cognition, motor symptoms, or quality of life. However, a years-long study of 787 Chinese PD patients suggested that MetS increased risk of cognitive deterioration [10]. In a large US study, PD patients with MetS showed a substantially higher annual growth in total and motor UPDRS scores compared to their counterparts without MetS [11]. Similar results were observed in a study on 1563 patients with mild Parkinsonian symptoms who completed a 6-year follow-up [19]. Our results, in agreement with these reports and the bulk of evidence, leads us to hypothesize that MetS may contribute to impair dopaminergic neurotransmission in the substantia nigra.

MetS and PD share some pathophysiological traits, such as the inflammatory and oxidative environment and insulin resistance [12]. Insulin resistance stimulates adipose tissue growth, hence proinflammatory adipokine secretion. A similar process has been suggested to occur in the brain. Chronic mild inflammation and a systemic oxidative milieu are concurrent. Neuroinflammation may cause reductions in neurotrophic factors, leading to neurodegeneration. The glucagon-like peptide-1 (GLP-1) receptor agonist exenatide, used in Diabetes Mellitus Type 2, has shown promising results for motor symptoms in PD clinical trials [20]. These results emphasize the importance of insulin resistance in the genesis of motor dysfunction in PD. Extracellular vesicles produced in adipocytes are able to cross the brain–blood barrier and contribute to neuroinflammation in MetS [7]. Candesartan inhibition of their proinflammatory effects [8] stresses the importance of tissue Renin–Angiotensin Systems intertwined in metabolic and neurodegenerative diseases [21,22]. Several pieces of evidence suggest that Heat Shock Proteins (Hsp) might represent a link between PD and MetS as well [23,24,25,26]. Finally, insulin/IGF1, TOR, AMPK, and sirtuin pathways have been involved in proteostasis control [27] and might also provide a link between MetS and PD [28,29,30,31].

The genetic traits connecting MetS and PD remain unknown. In our study, we compared the frequency of the 72 SNPs known to modify the risk of PD [13] among patients with or without MetS. MetS was associated with a higher frequency of the ZNF646.KAT8.BCKDK_rs14235 variant and a lower frequency of the variants NUCKS1_rs823118 and CTSB_rs1293298. The BCKDK gene codifies a branched-chain ketoacid dehydrogenase kinase, which participates in lipid metabolism, and its mutation has been associated with nonalcoholic fatty liver disease, cancer [32,33], PD, heart failure, neurodevelopmental disorders [13], and other conditions. In the brain, the BCKDK_rs14235 variant is related to multiple genes like VKORC1, KAT8, BCKDK, and ZNF646. Interestingly, the CTSB, ZNF646, and KAT8 genes have been associated with obesity [34,35]. The BCKDK_rs14235 variant increases the risk of PD, perhaps by modifying KAT8 gene expression, which is involved in modulating autophagic flux [36].

NUCKS1 has been linked to transcription modulation and chromatin architecture, cell cycle regulation, and DNA repair [27]. It is largely expressed in stem cells and the brain and appears in connection with MetS, PD, and cancer [27]. The NUCKS1 protein binds to DNA and nucleosomes and causes structural chromatin modifications [28]. Therefore, this multifaceted NUCKS1 protein regulates several signal transduction pathways and biological processes.

CTSB codes for cathepsin B, a lysosomal cysteine protease of the C1 family of peptidases. Alternative splicing generates the cathepsin B protein. Its endopeptidase and exopeptidase activities might be involved in protein turnover [29]. Cathepsin B participation in lysosomal α-synuclein degradation [30] and its contribution to PD risk [13] have been reported. In obese patients, white adiposity leads to cathepsin B release and adipocyte hypertrophy [31]. Adipocyte autophagy, macrophage infiltration, and inflammation induced by cathepsin B contribute to MetS [31].

This study has several limitations that should be acknowledged. First, the observational nature of the PPMI study prevents any causal inference between metabolic syndrome and Parkinson’s disease progression. Second, MetS status was assessed only at baseline, and potential changes in metabolic parameters during follow-up were not considered. Third, the relatively small number of patients with MetS may have limited the statistical power for some genetic and clinical comparisons. Additionally, although multiple covariates were adjusted for, the possibility of residual or unmeasured confounding cannot be entirely excluded. Lastly, as this study relied on a well-characterized research cohort, the generalizability of findings to broader or more heterogeneous populations may be limited.

Our findings support the existence of a shared genetic background between Parkinson’s disease (PD) and metabolic syndrome (MetS), reinforcing the hypothesis that metabolic alterations may modulate the progression of PD. Given the observed association with more severe motor symptoms, MetS may serve as a relevant modifier of disease phenotype. These results underscore the importance of early identification and close clinical monitoring of PD patients with MetS and highlight the need for further research to clarify the molecular mechanisms underlying this relationship.

## 5. Conclusions

This study highlights a significant clinical and genetic link between metabolic syndrome (MetS) and Parkinson’s disease (PD). We demonstrated that PD patients with comorbid MetS present more severe motor symptoms throughout the disease course, with consistently higher MDS-UPDRS Part III scores over a 5-year follow-up. These findings portray MetS as a disease modifier, contributing to the acceleration of motor symptom progression in PD.

At the genetic level, our analysis revealed differential frequencies of specific SNPs in patients with and without MetS. The increased prevalence of the ZNF646.KAT8.BCKDK_rs14235 variant in MetS patients implies a potential role for genes implicated in lipid metabolism and autophagic regulation at the intersection of metabolic and neurodegenerative pathways. Conversely, the decreased frequency of NUCKS1_rs823118 and CTSB_rs1293298 variants in MetS patients suggests a possible protective effect conferred by pathways involved in chromatin remodelling and lysosomal protein turnover.

In sum, our findings support the premise of a shared molecular basis between PD and MetS, involving convergent pathogenic mechanisms such as insulin resistance, neuroinflammation, oxidative stress, and defective proteostasis. Notably, our findings emphasize the importance of early detection of MetS in PD patients, as it may provide prognostic insight and facilitate tailored clinical monitoring and therapeutic approaches.

Future research involving larger, more diverse cohorts is essential to validate these associations and to determine whether metabolic interventions could attenuate PD progression in individuals with comorbid MetS.

## Figures and Tables

**Table 1:** Baseline characteristics of PD patients with and without metabolic syndrome.

	PD + MetS (*n* = 34)	PD–MetS (*n* = 389)	*p*-Value
Patients’ characteristics			
Male sex	32 (94%)	246 (63%)	<0.01
Age	64.9 ± 9.4	61.4 ± 9.7	0.04
PD family history	8 (24%)	126 (32%)	0.38
Hoehn and Yahr score			
I	8 (24%)	177 (46%)	0.01
II	26 (76%)	212 (54%)	
MDS-UPDRS Total score	30.7 ± 9.6	26.5 ± 11.2	0.03
MetS components			
Body mass Index	29 (85%)	241 (62%)	<0.01
Hyperglycemia	17 (50%)	5 (1%)	<0.01
Low HDL-C	13 (38%)	23 (6%)	<0.01
Hypertriglyceridemia	19 (56%)	11 (3%)	<0.01
Hypertension	28 (83%)	255 (66%)	0.02

MetS: metabolic syndrome. Means ± SD are shown.

**Table 2:** MDS-UPDRS subscores during the follow-up period.

Year	Part I		Part II		Part III		Part IV	
	PD–MetS	PD + MetS	PD–MetS	PD + MetS	PD–MetS	PD + MetS	PD–MetS	PD + MetS
0	1.2 ± 1.6	1.1 ± 1.2	5.8 ± 4.1	7.1 ± 4.7 **	20.7 ± 9.0	23.6 ± 7.2 **	-	-
1	1.5 ± 1.8	1.8 ± 1.7	7.2 ± 5.0	9.4 ± 4.5 *	24.2 ± 10.4	28.4 ± 10.0 *	0.2 ± 0.9	0.5 ± 1.3
2	1.9 ± 2.3	1.9 ± 2.0	7.8 ± 5.3	9.4 ± 5.2	26.4 ± 11.2	34.8 ± 10.7 **	0.5 ± 1.4	0.6 ± 1.7
3	1.9 ± 2.3	2.3 ± 1.9	8.7 ± 5.7	10.4 ± 5.5	27.7 ± 12.1	35.6 ± 9.9 **	0.7 ± 1.7	1.1 ± 2.1
4	2.2 ± 2.5	2.5 ± 3.6	9.7 ± 6.7	11.8 ± 5.5	30.4 ± 12.8	35.2 ± 9.7 *	1.4 ± 2.5	1.4 ± 2.0
5	2.3 ± 3.0	2.3 ± 2.0	9.9 ± 6.8	11.7 ± 5.9	29.9 ± 13.6	35.7 ± 9.5 *	2.0 ± 2.8	1.8 ± 2.0

MetS = metabolic syndrome. Means ± SD are shown. * *p* < 0.05, ** *p* < 0.01 (*t*-test) vs. PD + MetS.

**Table 3:** SNPs associated with MetS in PD.

Number of Risk Alleles for Each SNP	Overall	No MetS	MetS	Chi-Sq	Additive Model	Dominant Model	Recessive Model	Full Model
	(N = 409)	(N = 376)	(N = 33)	*p*-Value	OR (95% CI)	OR (95% CI)	OR (95% CI)	OR (95% CI)
					*p*-Value (AIC)	*p*-Value (AIC)	*p*-Value (AIC)	*p*-Value
GBA_N370S_rs76763715								
0	401 (98.0%)	370 (98.4%)	31 (93.9%)	0.003	4.22 (0.99, 16.19)	3.98 (0.57, 18.12)	-	2.91 (0.61, 12.70)
1	7 (1.7%)	6 (1.6%)	1 (3.0%)		0.03 (229.6)	0.10 (231.25)		0.141
2	1 (0.2%)	0 (0%)	1 (3.0%)		*SELECTED*			
NUCKS1_rs823118								
0	135 (33.0%)	128 (34.0%)	7 (21.2%)	0.011	0.98 (0.59, 1.62)	1.92 (0.85, 4.90)	0.26 (0.04, 0.88)	0.21 (0.03, 0.77)
1	197 (48.2%)	173 (46.0%)	24 (72.7%)		0.93 (233.29)	0.14 (203.97)	0.05 (228.57)	0.043
2	77 (18.8%)	75 (19.9%)	2 (6.1%)				*SELECTED*	
CTSB_rs1293298								
0	237 (57.9%)	211 (56.1%)	26 (78.8%)	0.027	0.62 (0.38, 1.01)	0.34 (0.13, 0.77)	*-*	0.35 (0.13, 0.84)
1	143 (35.0%)	136 (36.2%)	7 (21.2%)		0.06 (229.75)	0.01 (226.49)		0.025
2	29 (7.1%)	29 (7.7%)	0 (0%)			*SELECTED*		
ZNF646.KAT8.BCKDK_rs14235								
0	147 (35.9%)	134 (35.6%)	13 (39.4%)	0.023	1.29 (0.77, 2.14)	0.85 (0.41, 1.81)	2.59 (1.12, 5.62)	3.06 (1.24, 7.29)
1	198 (48.4%)	188 (50.0%)	10 (30.3%)		0.33 (232.46)	0.67 (233.21)	0.02 (228.48)	0.012
2	64 (15.6%)	54 (14.4%)	10 (30.3%)				*SELECTED*	
COMT_rs4633								
0	116 (28.4%)	104 (27.7%)	12 (36.4%)	0.105	0.62 (0.38, 1.01)	0.67 (0.32, 1.45)	0.33 (0.10, 0.87)	0.40 (0.11, 1.10)
1	179 (43.8%)	162 (43.1%)	17 (51.5%)		0.06 (229.75)	0.29 (232.22)	0.04 (228.25)	0.107
2	114 (27.9%)	110 (29.3%)	4 (12.1%)				*SELECTED*	

The Akaike Information Coefficient (AIC) was used to compare the additive, dominant, and recessive models. The one with the lowest AIC was selected for the multivariate model, provided the *p*-value was <0.15.

## Data Availability

Data used in the preparation of this article were obtained from the Parkinson’s Progression Markers Initiative (PPMI) database (www.ppmi-info.org/data (accessed on 10 November 2020)). For up-to-date information on the study, visit www.ppmi-info.org.
